# Howler monkey (*Alouatta palliata*) thermal behavioral strategies and resting time are temperature-dependent

**DOI:** 10.1093/cz/zoaf068

**Published:** 2025-10-22

**Authors:** Rael Martín Palestino-Sánchez, N Sofía Huerta-Pacheco, Juan Francisco Rodríguez-Landa, María Fernanda López-Flores, Francisco García-Orduña, María de Jesús Rovirosa-Hernández

**Affiliations:** Doctorado en Neuroetología, Instituto de Neuroetología, Universidad Veracruzana, Xalapa, Veracruz, CP 91190, México; SECIHTI, Escuela Nacional de Ciencias Forenses, Universidad Nacional Autónoma de México, CDMX, CP 04510, México; Instituto de Neuroetología, Universidad Veracruzana, Xalapa, Veracruz, CP 91190, México; Doctorado en Ciencias Biomédicas, Centro de Investigaciones Biomédicas, Universidad Veracruzana, Xalapa, Veracruz, CP 91190, México; Instituto de Neuroetología, Universidad Veracruzana, Xalapa, Veracruz, CP 91190, México; Instituto de Neuroetología, Universidad Veracruzana, Xalapa, Veracruz, CP 91190, México

**Keywords:** ambient temperature, behavioral postures, primates, thermoregulation, tree stratum

## Abstract

Primates exhibit thermal behaviors (use of postures in tree stratum) that help regulate body temperature in response to environmental conditions, reducing the energetic and water costs of thermoregulation. This study examined the relationship between ambient temperature and thermal behavioral strategies during resting periods in mantled howler monkeys (*Alouatta palliata*) across 3 sites in Veracruz, Mexico: Agaltepec (AGA; 28.4 ± 4.7 °C), Mirador Pilapa (MP; 28.8 ± 2.1 °C), and Zapoapan (ZAP; 28.9 ± 2.5 °C). We used focal-animal sampling, randomly selecting individuals and recording the time invested in each posture and the tree stratum in which they rested, and the ambient temperature. All statistical analyses were performed independently between sites. The results indicate that, across the 3 study sites, a consistent pattern of posture adopted within specific tree strata was observed, despite differences in altitude, vegetation, and ambient temperature. We found a strong positive correlation between lower temperatures and the time recorded in the semi-fetal posture in the high strata (Rc > 0.7547), and between higher temperatures and the time recorded in the extended posture in the low strata (Rc > 0.6803). These results suggest howler monkeys combine posture and vertical positioning to optimize thermoregulation. However, as temperatures rise, the preference for extended postures in lower strata—where temperatures may be cooler—leads to longer rest periods. This behavioral shift could reduce feeding and hydration, potentially increasing the risk of dehydration or heat stress under future climate conditions.

## Graphical Abstract

**Graphical Abstract:**
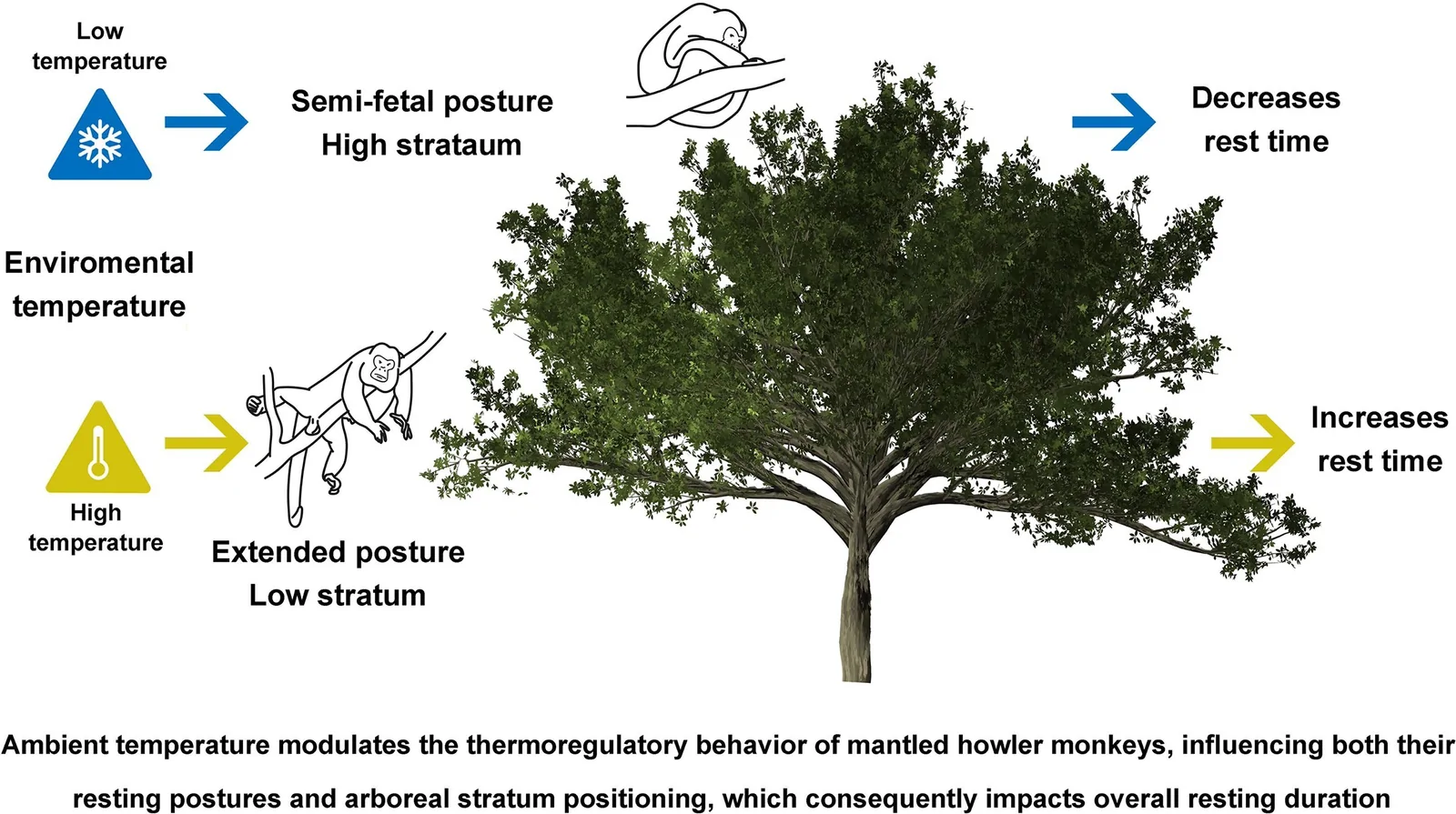

Increased anthropogenic activities have had an irreversible impact to the climate, leading to an accelerated increase in global ambient temperatures (Haustein et al. 2017; IPCC 2023). This rapid change poses a serious threat to biodiversity, particularly for species inhabiting small forest fragments, where reduced vegetation cover and steeper thermal gradients exacerbate thermal stress (Ewers and Banks-Leite 2013; Hofmeister et al. 2019). These altered conditions affecting species’ biology and forcing them to live in unfavorable thermal conditions (Pecl et al. 2017; Sales et al. 2019; Fuller et al. 2021). To cope with these changes, mammals must adapt by employing strategies that help them to maintain thermal balance with the increasing environmental changes (Robertshaw 2006; Collier and Gebremedhin 2015; Mitchell et al. 2018; Shelton and Alberts 2018; Fuller et al. 2021).

Mammals regulate their body temperature through physiological processes such as transpiration or thermogenesis (Rhoades and Bell 2012; Tattersall et al. 2012; Collier and Gebremedhin 2015), however, these processes require water and energy (Tattersall et al. 2012; Mitchell et al. 2018). When these resources are limited, mammals often resort to behavioral thermoregulation to reduce physiological costs (Mitchell et al. 2018; Fuller et al. 2021). These thermal behaviors include adjusting rest period according to thermal conditions, adopting specific postures, or selecting particular sites during rest; these strategies can facilitate conservation, gain, or dissipate heat (Eppley et al. 2017; Fuller et al. 2021; Hilário et al. 2022; Giroux et al. 2023).

Non-human primates (hereafter “primates”) rely heavily on behavioral thermoregulation, as they exhibit limited use of physiological mechanisms such as sweating or panting (Best and Kamilar 2018; Thompson and Hermann 2024). Consequently, they maintain thermal balance primarily through behavioral adjustments, including shifts in activity budgets, evaporative cooling, posture changes, and microhabitat use (Paterson 1981; Bicca-Marques and Calegaro-Marques 1998; Gestich et al. 2014; Thompson et al. 2016; Palestino-Sánchez et al. 2020; Brividoro et al. 2023; Chen-Kraus et al. 2023; Thompson and Hermann 2024). Primates often combine behavioral thermal strategies. For example, they may pair specific postures with particular arboreal strata to regulate body temperature. In colder conditions, primates adopt compact postures (e.g., curled or semi-fetal) and occupy sun-exposed strata to conserve or generate heat (Bicca-Marques and Calegaro-Marques 1998; Gestich et al. 2014; Palestino-Sánchez et al. 2020). In contrast, under warm conditions, they adopt extended postures—spreading their limbs away from the body—and select shaded microhabitats to facilitate heat dissipation and reduce thermal load (Bicca-Marques and Calegaro-Marques 1998; Palestino-Sánchez et al. 2020; Brividoro et al. 2023).

In mantled howler monkeys (*Alouatta palliata*), studies have documented the use of different postures (Paterson 1981; Thompson et al. 2016; Palestino-Sánchez et al. 2020) and different arboreal strata (Thompson et al. 2016) during resting periods. These behavioral thermoregulation strategies vary with ambient temperature (Thompson et al. 2016; Palestino-Sánchez et al. 2020). However, it remains unclear whether mantled howler monkeys invest equal time in each strategy or whether posture choice correlates with specific tree strata. Understanding these relationships may clarify how these monkeys regulate their body temperature in response to ambient conditions through thermal behavior. In this study, we tested the hypothesis that *A. palliata* inhabiting in 3 small forest fragments adjusts resting time in relation to posture and tree stratum as a response to ambient temperature. Specifically, we predicted that, under warm conditions, resting duration would increase when individuals adopted an extended posture in the lower strata. This prediction describes the interdependence between thermal behavior, resting time, and ambient temperature, suggesting that increased resting time functions as a behavioral strategy to reduce endogenous heat production and buffer the effects of heat stress.

## Materials and methods

### Study site and subjects

**Figure 1:**
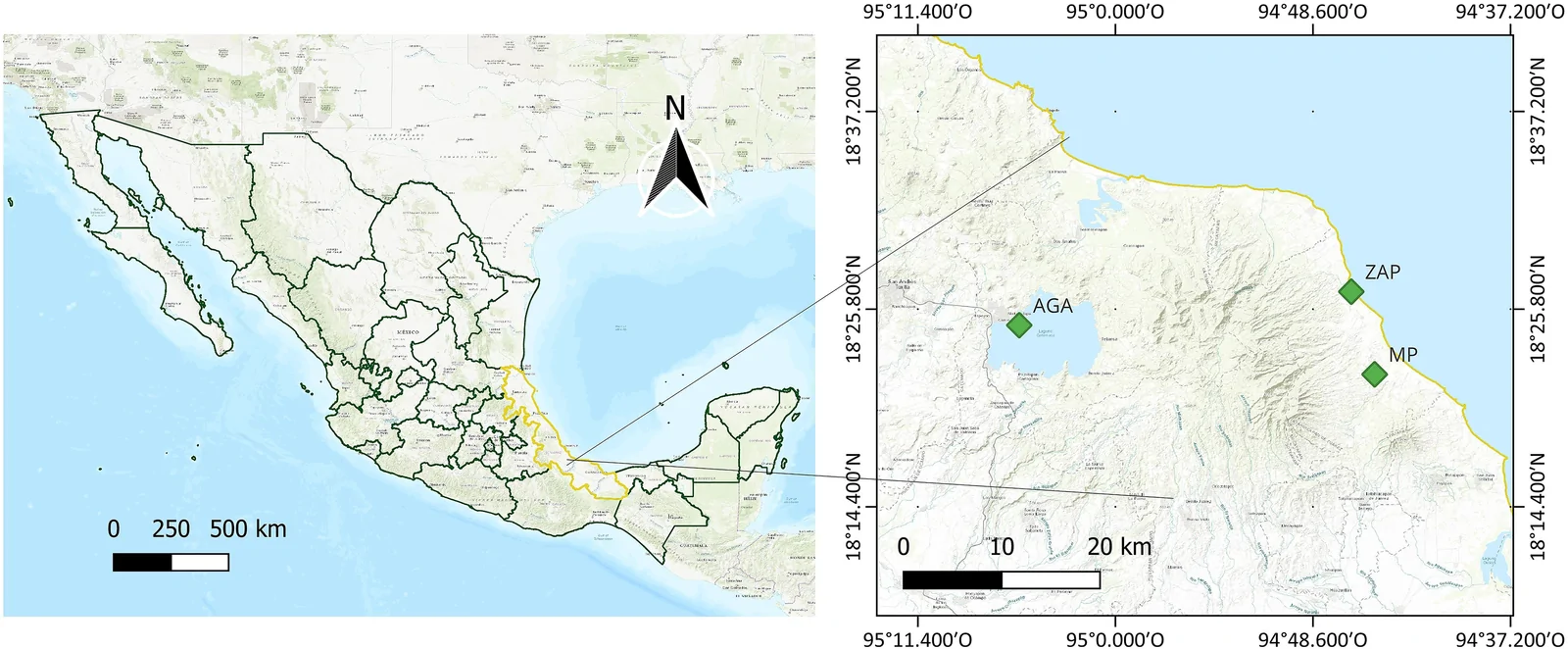
Map of the 3 study sites [Agaltepec (AGA); Mirador Pilapa (MP); Zapoapan (ZAP)] in the region of Los Tuxtlas, Veracruz, Mexico. The map was created using QGIS version 3.2023.2 Firenze (2023) with the EPSG:4326—WGS 84 coordinate reference system. The map features an XYZ tile layer from OpenStreetMap, as well as vector layers of national and state boundaries from INEGI (2022). The sampling points (AGA, ZAP, MP) are based on geographic coordinates collected by the authors for this study. The basemap is credited to OpenStreetMap, which is licensed under the Open Database License (ODbL). The design was created by Sandra Cecilia Uribe Barrera using QGIS exclusively for academic and non-commercial use.

This study was conducted in 3 different sites located in the region of Los Tuxtlas (Fig. 1). The first site, Agaltepec Island (AGA), is located in the municipality of Catemaco, Veracruz, Mexico, between (18°24′−18°25′ N and 95°05′−95°06′ W), covering an area of 8.3 ha, and to 340 masl. Four vegetation types are present on the AGA: medium subdeciduous forest, secondary vegetation, riparian vegetation, and grassland (López-Galindo and Acosta-Pérez 1998). The area surrounding the site was subject to human activities, including fishing and tourism.

In Catemaco there is a mean annual temperature of 25.2 °C, with a mean annual minimum and maximum range of 17.1–33.3 °C (00030022 monitoring station of the National Meteorological System, located at coordinates 18°25′10″ N, 95°06′49″ W; climatic station of La Flor de Catemaco, S. de R.L. de C.V.).

The second site, Mirador Pilapa (MP), between (18°21′55.4′′N, 094°44′50.6′′W), covering an area of 19.8 ha, and to 115 masl. In MP, the main vegetation is represented by tall evergreen forest, medium subdeciduous forest, and secondary vegetation (Guevara et al. 2004; Vázquez-Torres et al. 2010). This site is located in a fragment ≤10 m from areas adjacent to the town.

The third site, Zapoapan (ZAP) between (18°26′56.2′′N, 094°46′31.5′′W), covering an area of 13.8 ha, and 8 masl. In ZAP, the vegetation is mainly represented by medium subdeciduous forest, and to a lesser extent by tall evergreen forest, secondary vegetation, and grassland (Guevara et al. 2004; Vázquez-Torres et al. 2010). This site is located in a fragment that is ≤20 m from areas where human activities such as livestock farming and fishing are carried out.

The second and third sites are within the municipality of Tatahuicapan of Juárez, Veracruz, Mexico, southeast of the Sierra of Santa Martha. In Tatahuicapan of Juárez there is a mean annual temperature of 26.4 °C, and a mean minimum and maximum range of 21 °C to 31 °C (National Institute for Federalism and Municipal Development [INAFED], 2022; Weather station Minatitlan Airport, Veracruz, Mexico 1994–2023 [18°6′ 12.312″N, 94°34′50.452″W], 2023).

Across the 3 study sites, natural predators were absent. Nevertheless, the proximity to human settlements and domestic animals poses potential risks, particularly from feral dogs, which are known to attack howler monkeys (Cristóbal-Azkarate et al 2015).

Three different groups with different individuals of mantled howler monkeys (*Alouatta palliata*) were observed; this species has diurnal habitats, with a high investment of time in resting, between 60% and 80% of their activity (Serio-Silva 1995; Milton 1998; Estrada et al. 1999; Asensio et al. 2007; Cristóbal-Askarate and Arroyo-Rodríguez 2007; Schreier et al. 2021). The group composition by site was as follows: At the AGA site, we observed 17 individuals (6 adult females, 7 adult males, 1 sub-adult female, and 3 sub-adult males); at the MP site, we observed 9 individuals (4 adult females, 4 adult males, and 1 juvenile); and at the ZAP site, we observed 8 individuals (4 adult females, 3 adult males, and 1 juvenile).

### Behavioral records

This study included only random observations between individuals in each of the groups, with non-overlapping individuals across sites—those capable of performing behaviors (e.g., feeding) without external aid—including adults (excluding females with infants), sub-adults, and juveniles, which were identified following the descriptions proposed by Balcells and Veà (2009).

The duration and frequency of each thermoregulatory behavioral strategy were recorded using focal animal sampling (Altmann 1974) during resting periods, defined as times when individuals were inactive or adopting postures unrelated to other behaviors (e.g., feeding). We make the observations randomly between adult female, adult male, sub-adult female, sub-adult male, and juvenile, considering recording all individuals per day in each site. At AGA, because focal samples lasted 60 min, combined with the limited observation hours and the relatively large group size (*n* = 17), it was not possible to record all individuals each day. Instead, we ensured that each individual was sampled at least once per month.

Although the observations in this study were conducted on different time scales—which should be considered a limitation due to their potential effect on the results—data at each site were collected over several months and years (8 months at AGA and 10 months at MP and ZAP). Beyond intermediate months, the dataset also encompassed the warmest (April and May) and the coldest (January) months in the Los Tuxtlas region across the 3 sites, which were particularly important for evaluating the influence of temperature on thermal behavior.

For the AGA site, data were collected during 60-min focal samples across 8 months between the period of October 2018—August 2019, 6 days per month, from 07:00 to 15:00 h. A total of 384 h of fieldwork were obtained, with a mean ± SD of hours recorded during resting periods per individual of 5.6 ± 2.8 h. For MP and ZAP sites, we conducted 10-min focal samples over 10 months from June 2022 to July 2023, 8 days per month (4 per site), from 06:00 to 17:00 h. A total of 440 h of fieldwork per site, with a mean ± SD of hours recorded during resting periods per individual of MP 32.6 ± 13.3 h, and ZAP 40.4 ± 16.8 h.

Although the observation times at AGA differed from those at MP and ZAP—a limitation that could affect both data collection and interpretation—we consider that the ambient temperature data were still representative for analyzing the relationship between environmental conditions and thermal behavior. This is because measurements at all sites were taken during key periods of the day: the onset of morning sunlight (07:00 h, at AGA; 06:00 h, at MP and ZAP), the midday peak (around 12:00 h), and the initial cooling phase in the afternoon (approximately 15:00 h). The earlier ending time at AGA represents a limitation, and results for this site should therefore be interpreted with caution.

**Figure 2:**
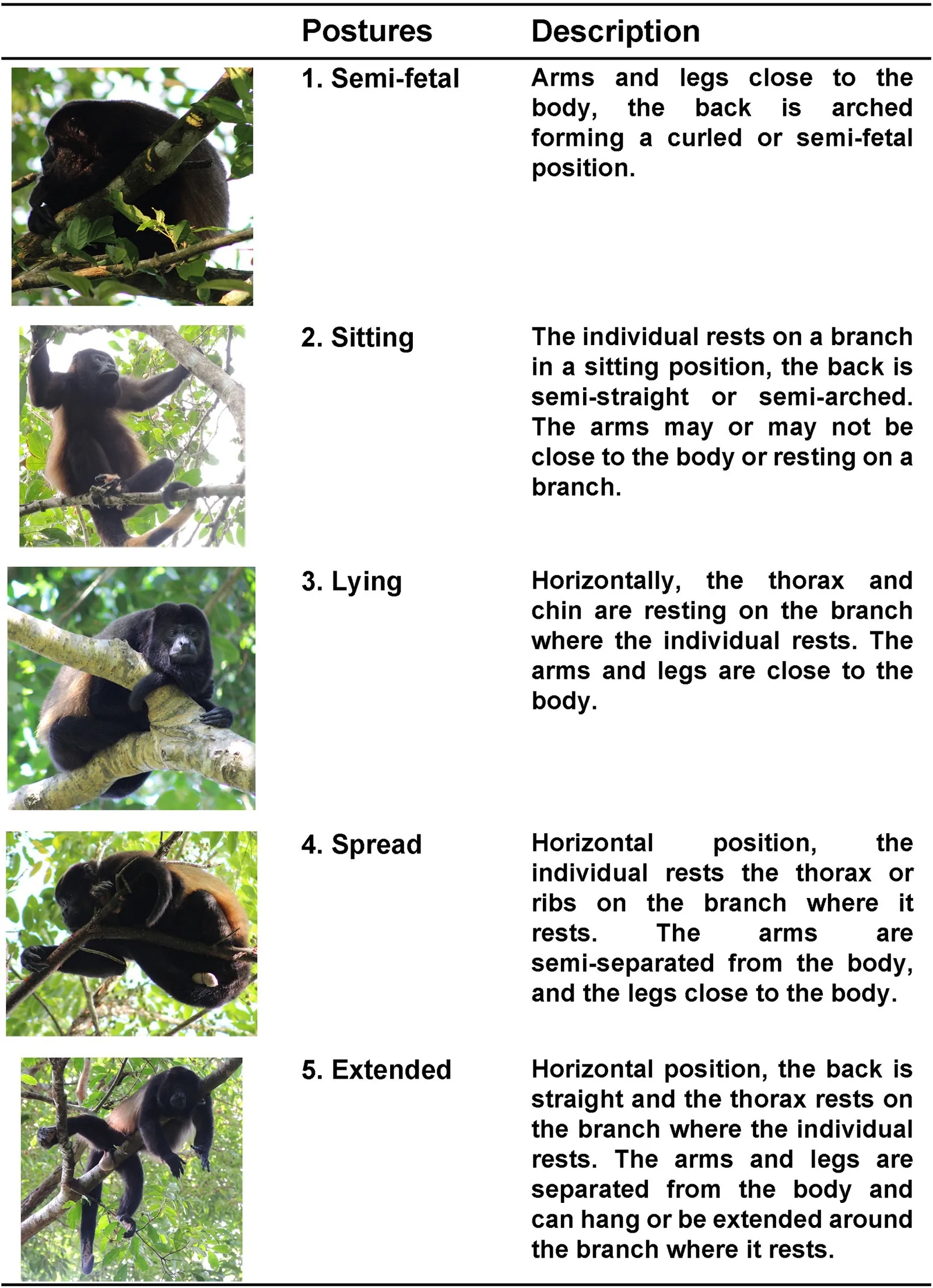
Ethogram to identify and record the body postures adopted by *A. palliata* during rest (Paterson 1981; Bicca-Marques and Calegaro-Marques 1998; Palestino-Sánchez et al. 2020).

**Figure 3:**
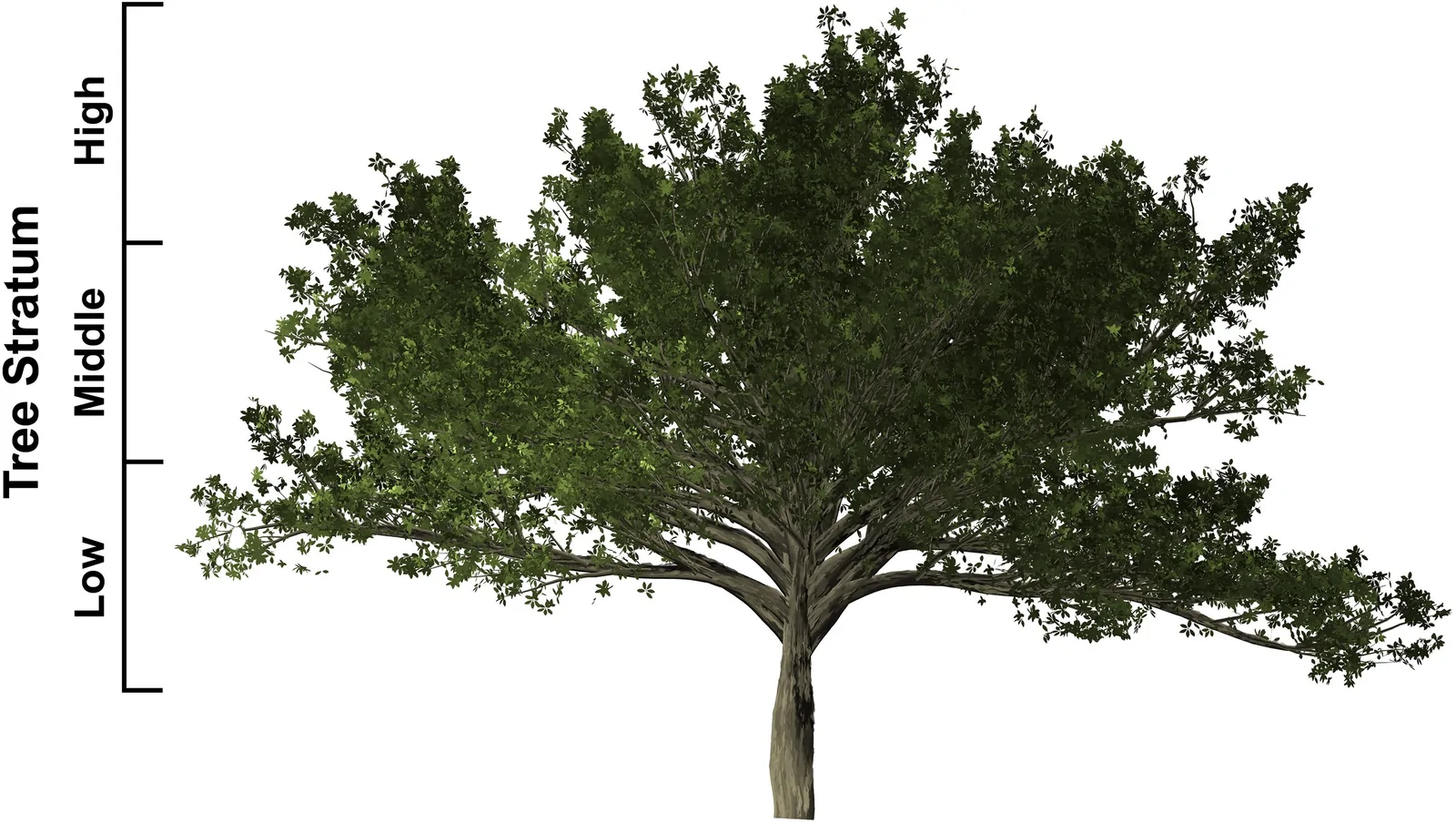
Description of the classification of tree stratum used by *A. palliata* during rest (Urbani 2003; Aristizabal et al. 2018).

During the focal animal observations, the posture adopted by howler monkeys when resting was observed (Fig. 2). These postures were identified based on previous descriptions reported by Paterson (1981); Bicca-Marques and Calegaro-Marques (1998), and Palestino-Sánchez et al. (2020). In addition, the tree stratum where howler monkeys rest and adopt each posture was recorded, for this, the classification described by Urbani (2003), and Aristizabal et al. (2018) was used. This classification used by *A. palliata*, which consists of dividing the tree area empirically from the lower branches to the upper branches, dividing the area into 3 strata: High, medium, and low (Fig. 3), this classification was performed individually for each tree where mantled howler monkeys were observed resting, due to the considerable variation in tree height across sites—ranging from 3 to over 20 meters. To control for inter-observer variability when recording tree stratum use, the observer was trained using a standardized protocol based on visual estimation. Control sessions were conducted before data collection to ensure consistency in stratum classification.

### Ambient temperature record

The ambient temperature was measured using a portable weather station (Kestrel® 3000, Global Test Supply, Wilmington, NC), which has an accuracy of ± 0.5 °C. This data was obtained for each of the sites at the beginning of each behavioral observation or when there was a change in the posture or tree stratum, at a height of ≈ 2 meters, separating the portable weather station from the observer's body to prevent any interference with the data recording, and as close as possible to the location of the study subjects (<10 meters). This method has inherent limitations because it offers only an approximation of ambient temperature and does not reflect the variation between the different heights where howler monkeys stayed.

### Data analysis

All statistical analyses were conducted based on the following points: (1) identify normal distribution of the data, (2) compare the sites considering time rest and temperature, (3) compare time rest and temperature of the posture and arboreal stratum for each site, and finally (4) identify the association of the sample for each site.

To evaluate data distribution, we first applied the Shapiro–Wilk tests (Hanusz et al. 2016) of the numeric variables (time rest and temperature). Since the assumption of normality was not met, non-parametric tests were used in the subsequent steps.

Kruskal-Wallis test was applied to compare ambient temperature (°C) and mean of rest time between each site, to assess whether significant differences existed across groups/sites. Under the hypothesis, if at least 1 group/site differed from the others, they would be independent of each other; this was corroborated with a post hoc pairwise test. The sites differed significantly in ambient temperature (*P* < 0.05) and time (*P* < 0.05), confirming that they represent different behavior (independence) for each sample.

Additionally, we evaluated the influence of the sex of the quantitative variables; the result confirmed (*P* > 0.05) the exclusion of this factor in the analysis based on prior evidence indicating no significant sex-related differences in thermoregulatory behavior (Palestino-Sánchez et al. 2020).

After confirming the independence of the samples by group/sites, the Kruskal-Wallis test was applied to compare the mean ambient temperature (°C) and the mean time spent (minutes) across posture and arboreal stratum categories during resting periods by site, using a significance threshold of *P* < 0.05 (McKnight and Najab 2010).

Since body posture may be associated with specific tree strata, we used canonical correlation analysis (CCA), to examine the relationship between posture-specific resting time across tree strata and ambient temperature. A data matrix was constructed using mean values that represented each thermoregulatory behavioral strategy. Set 1 included the mean ambient temperature recorded for each posture–stratum combination, while Set 2 comprised the corresponding mean resting time (see Supplementary Appendix A). To ensure statistical independence of observations and avoid the problem of pseudoreplication (Hurlbert 1984), mean values were calculated from repeated measurements within each experimental unit (group/site) This approach reduces within-unit variability but provides more conservative and statistically robust estimates, while meeting the assumption of independence required for CCA and other multivariable methods (Legendre and Legendre 2012). This research applies a CCA to identify a linear combination (canonical variables) that maximizes the correlation between 2 sets of variables (Hardoon et al. 2004), enabling us to assess the associations between posture-specific resting time, ambient temperature, and arboreal stratum.

All analyses were performed in RStudio Team (2024) using the cancor function by the CCA (Gonzáles and Dejean 2023) and vegan (Oksanen et al. 2024) packages.

## Results

### Thermal behavioral strategies

We observed that despite studying 3 locations with different individuals, the same thermal behavioral pattern occurred at all sites. Howler monkeys adopted specific body posture according to the mean temperature specific body postures within particular arboreal strata (Table 1). During rest, differences were observed in the mean time spent across postures and arboreal stratum, with the semi-fetal and extended postures exhibiting the highest mean durations (Table 2). Specifically, mantled howler monkeys tended to adopted the semi-fetal posture in the high stratum at lower temperatures, and they adopted the extended posture in the low stratum at higher temperatures.

**Table 1:** Number of records, temperature range, and mean ambient temperature recorded in each body posture in each tree strata for each site.

		Posture (Mean ± SD)				
Site	Stratum	Semi-fetal °C	Sitting °C	Lying °C	Spread °C	Extended °C
Agaltepec (**AGA**)	High	20.78 ± 3.30 (*n* = 18)	25.18 ± 3.01 (*n* = 24)	26.23 ± 2.53 (*n* = 24)	28.61 ± 3.62 (*n* = 7)	—
	Middle	—	27.02 ± 3.69 (*n* = 12)	28.81 ± 2.51 (*n* = 21)	31.79 ± 2.64 (*n* = 16)	33.63 ± 2.42 (*n* = 22)
	Low	—	33.25 ± 0.07 (*n* = 2)	31.9 (*n* = 1)	32.27 ± 1.56 (*n* = 4)	35.65 ± 3.29 (*n* = 24)
	**Range (°C)**	**17.4–27.0**	**18.4–33.3**	**21.2–31.9**	**22.1–36.7**	**30.1–40.2**
Mirador Pilapa (MP)	High	27.12 ± 1.75 (*n* = 263)	28.10 ± 2.70 (*n* = 231)	28.63 ± 1.62 (*n* = 228)	29.65 ± 1.79 (*n* = 71)	—
	Middle	27.65 ± 1.73 (*n* = 21)	29.20 ± 1.67 (*n* = 165)	28.41 ± 1.73 (*n* = 167)	29.51 ± 1.28 (*n* = 293)	30.39 ± 1.00 (*n* = 49)
	Low	—	30.05 ± 1.26 (*n* = 20)	27.73 ± 4.11 (*n* = 3)	29.11 ± 2.49 (*n* = 20)	30.88 ± 1.52 (*n* = 163)
	**Range (°C)**	**21.1–31**	**21.4–35**	**23.9–32.9**	**24–33.7**	**29.2–35.5**
Zapoapan (**ZAP**)	High	24.83 ± 2.53 (*n* = 174)	28.81 ± 2.51 (*n* = 182)	27.96 ± 1.11 (*n* = 175)	29.77 ± 0.78 (*n* = 79)	—
	Middle	24.9 ± 4.52 (*n* = 2)	28.18 ± 2.74 (*n* = 183)	27.85 ± 1.31 (*n* = 82)	29.90 ± 1.09 (*n* = 264)	31.180 ± 1.04 (*n* = 169)
	Low	—	27.97 ± 2.17 (*n* = 42)	28.60 ± 1.13 (*n* = 2)	29.84 ± 1.22 (*n* = 41)	30.98 ± 1.22 (*n* = 241)
	**Range (°C)**	**19.6–30.2**	**19.3–34.8**	**24.1–30.1**	**26.9–32.5**	**29.2–38.1**

**Table 2:** Number of records and mean time recorded in each body posture in each tree strata for each site.

		Posture (Mean ± SD)				
Site	Arboreal stratum	Semi-fetal °C	Sitting °C	Lying °C	Spread °C	Extended °C
Agaltepec (AGA)	High	37.1 ± 15.4 (*n* = 18)	14.4 ± 8.5 (*n* = 24)	24.9 ± 13 (*n* = 24)	28.9 ± 13.3 (*n* = 7)	—
	Middle	—	18.6 ± 14.9 (*n* = 12)	24.9 ± 13 (*n* = 21)	31.1 ± 13.6 (*n* = 16)	49.2 ± 9.7 (*n* = 22)
	Low	—	12.1 ± 2.6 (*n* = 2)	60 (*n* = 1)	30.1 ± 5.7 (*n* = 4)	54.7 ± 9.1 (*n* = 24)
Mirador Pilapa (MP)	High	8.2 ± 2.6 (*n* = 263)	4.4 ± 3.3 (*n* = 231)	8.2 ± 2.6 (*n* = 228)	8.3 ± 2.3 (*n* = 71)	—
	Middle	8.1 ± 2.5 (*n* = 21)	4.2 ± 3.2 (*n* = 165)	8.8 ± 2.3 (*n* = 167)	8.6 ± 2.2 (*n* = 293)	8.9 ± 2.1 (*n* = 49)
	Low	—	4.3 ± 2.9 (*n* = 20)	7.5 ± 2.3 (*n* = 3)	8.5 ± 2.5 (*n* = 20)	9.2 ± 1.8 (*n* = 163)
Zapoapan (ZAP)	High	7.9 ± 2.8 (*n* = 174)	3.3 ± 2.8 (*n* = 182)	8.7 ± 2.3 (*n* = 175)	7.9 ± 2.7 (*n* = 79)	—
	Middle	7.3 ± 2.8 (*n* = 2)	3.8 ± 2.9 (*n* = 183)	8.6 ± 2.2 (*n* = 82)	8.7 ± 2.2 (*n* = 264)	9.1 ± 1.7 (*n* = 169)
	Low	—	3.1 ± 2.4 (*n* = 42)	8.8 ± 1.6 (*n* = 2)	8.2 ± 2.8 (*n* = 41)	9.3 ± 1.5 (*n* = 241)

### Comparison of body postures

In general, sitting, lying, and spreading postures were primarily observed in the middle stratum (Table 2). However, the posture in which mantled howler monkeys spent the most time resting was the extended posture in the low stratum (AGA: 54.7 ± 9.1 min; MP: 9.1 ± 1.7 min; ZAP: 9.3 ± 1.4 min; see Table 2), followed by the semi-fetal posture in the high stratum at AGA (37.1 ± 15.4 min), and the lying posture in the high and middle strata at MP (8.7 ± 2.2 min) and ZAP (8.8 ± 1.6 min). At all 3 study sites, sitting was the posture with the shortest duration recorded during resting in the low stratum (AGA: 12.0 ± 2.5 min; MP: 4.2 ± 2.9 min; ZAP: 3.0 ± 2.3 min).

**Figure 4:**
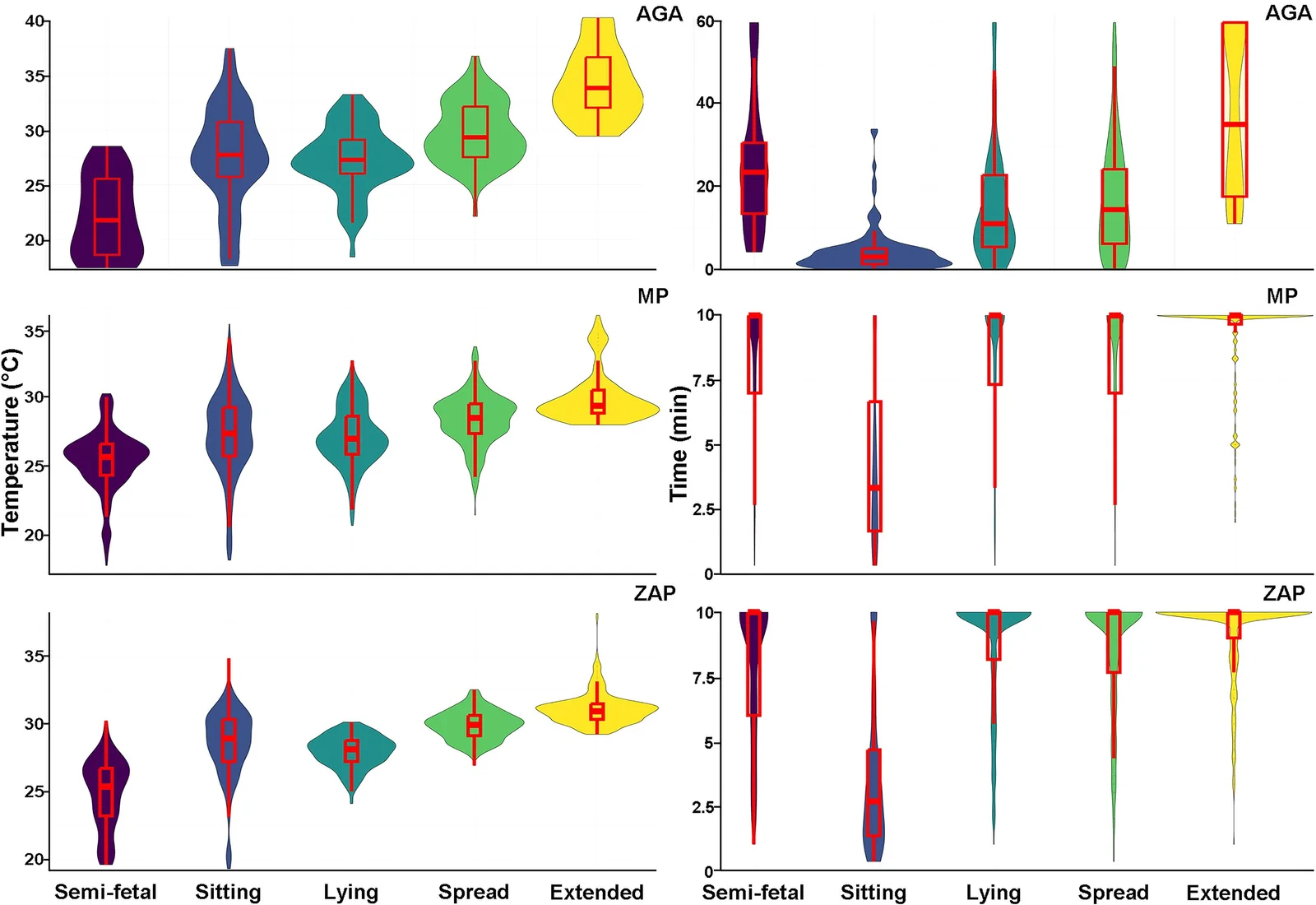
Comparison of the ambient temperature and time recorded in each of the body postures used by *A. palliata* during rest in the 3 sites.

Comparisons of ambient temperature recorded for each body posture showed differences across at each site (Fig. 4): AGA (*H* = 120.1, df = 4, *P* < 2.2e−16), MP (*H* = 460.34, df = 4, *P* < 2.2e−16), and ZAP (*H* = 899.08, df = 4, *P* < 2.2e−16). Likewise, differences were found in the time invested in each posture at each site (Fig. 4): AGA (*H* = 153.72, df = 4, *P* < 2.2e−16), MP (*H* = 481.55, df = 4, *P* < 2.2e16), and ZAP (*H* = 676.11, df = 4, *P* < 2.2e−16), but only the extended and semi-fetal postures showed differences in the recorded time compared with the others (see Supplementary Appendix B).

### Comparison of the tree stratum

**Figure 5:**
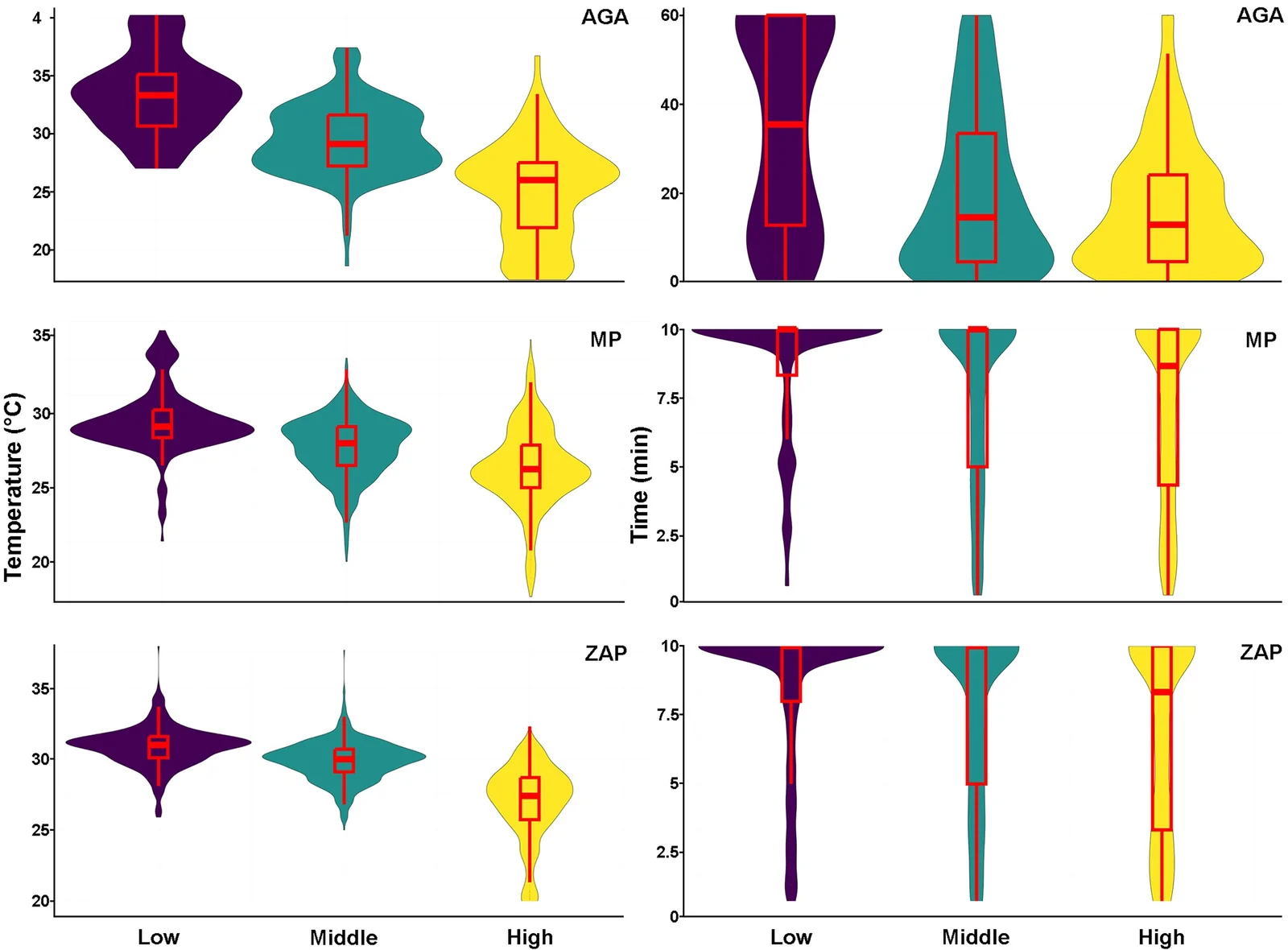
Comparison of the ambient temperature ranges and time were recorded at each site in each tree strata that uses *A. palliata* during rest.

The comparison showed that differences in ambient temperature were recorded in each tree stratum across the 3 sites (Fig. 5): AGA (*H* = 120.02, df = 2, *P* < 2.2e−16), MP (*H* = 291.86, df = 2, *P* < 2.2e−16), and ZAP (*H* = 364.41, df = 2, *P* < 2.2e−16). Similarly, differences were found in the time that the *A. palliata* spend resting in different tree strata (Fig. 5): AGA (*H* = 12.49, df = 2, *P* = 0.00194), MP (*H* = 41.69, df = 2, *P* < 8.813e−10), and ZAP (*H* = 48.12, df = 2, *P* < 3.544e−11). Additionally, we noted differences in rest time between the middle and high strata, but only in the fragment of AGA, there were no differences between the time reported in the middle and high stratum (Supplementary Appendix C).

### Correlation between posture and arboreal stratum

**Figure 6:**
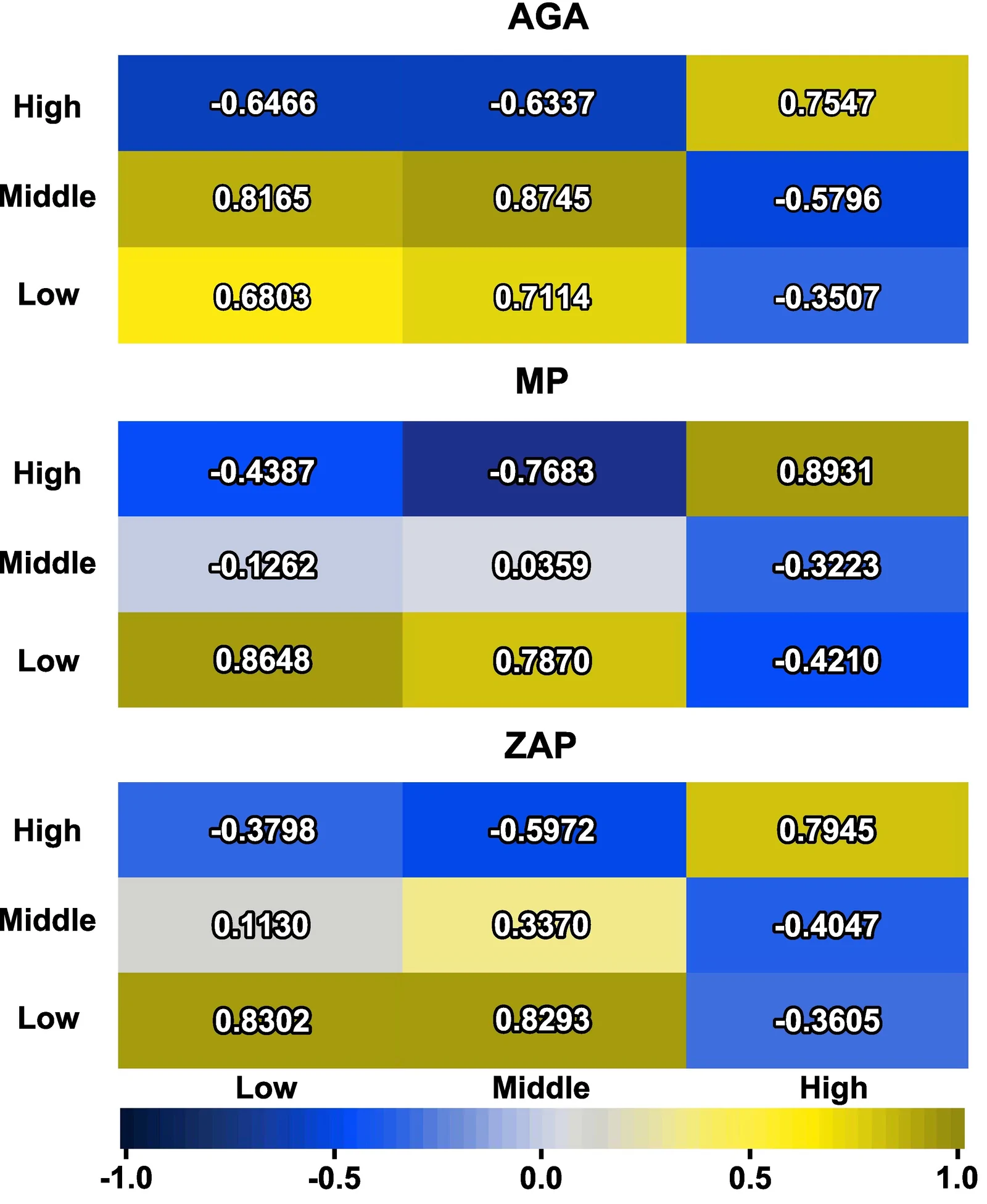
Canonical cross-correlation between the mean times and ambient temperatures associated with each posture in the tree stratum across study sites. Yellow indicates a positive cross-correlation, blue a negative one, and color intensity reflects the strength of the correlation.

The results showed that as the ambient temperature increased, the howler monkeys mainly used the extended posture in the lower tree strata. Conversely, when the ambient temperature was lower, they rested mainly in the upper tree stratum using the semi-fetal posture (Table 1). According to the CCA, there is a strong correlation between the ambient temperature and the time spent in each posture in every tree stratum for each site (Fig. 6).

At all 3 sites, a positive correlation (R_c_ > 0.6803) was observed in the “Low-Low” condition between the time spent in the extended posture and the temperature in the lower tree strata. Similarly, in the pattern we observed in the “High-High” condition, a positive correlation (R_c_ > 0.7547) was found between the time spent in the semi-fetal posture and the temperature in the high strata. This pattern was consistent across all sites.

The time spent in the extended posture is associated with the lower tree strata as the ambient temperature increases, while the time spent in the semi-fetal posture is associated with the high tree strata when the ambient temperature decreases. This relation is illustrated in the graphs obtained from the CCA (Supplementary Appendix D).

## Discussion

The present study described the relationship between the rest time, the use of thermal behaviors, and ambient temperature in *A. palliata*. Despite the unique characteristics of each site—such as vegetation, elevation, and temperature ranges (AGA: 17.4–40.2 °C; MP: 21.1–35.5 °C; ZAP: 19.3–38.1 °C)—mantled howler monkeys exhibited a consistent behavioral pattern. As ambient temperature increased, individuals adopted the extended posture in the lower stratum, and they rested for longer mean periods compared with other postures or when resting in other tree strata; these patterns suggest that posture and stratum are not used independently, but rather in conjunction, likely to maintain thermal balance with the environment.

Mammalian body size is a well-established factor influencing thermoregulation, primarily due to the surface area-to-volume ratio, which affects the rate of heat exchange per unit of body mass (Fuller et al. 2016, 2021). Given their relatively body size, mantled howler monkeys may be particularly susceptible to gaining heat from the environment. To mitigate this, they may reduce endogenous heat production by minimizing activity as temperatures rise (Terrien et al. 2011; Fuller et al. 2016, 2021). Because primates exhibit limited physiological thermoregulation—sweating, panting, and evaporative cooling are largely absent due to the scarcity of eccrine glands (Best and Kamilar 2018; Thompson and Hermann 2024)—they instead rely heavily on low-cost behavioral mechanisms. In mantled howler monkeys, the strategic combination of body posture and tree stratum during rest likely plays a central role in this process.

Previous research has documented various behavioral thermoregulation strategies in howler monkeys (Bicca-Marques and Calegaro-Marques 1998; Palestino-Sánchez et al. 2020; Brividoro et al. 2023; Thompson and Hermann 2024). While these strategies have often been considered independently, our findings reveal a positive correlation between posture and tree stratum use. Specifically, individuals adopted the semi-fetal posture in higher strata under cold conditions and the extended posture in lower strata during warmer conditions, but when mantled howler monkeys use thermal behaviors to dissipate heat, the time they rest tends to increase. This pattern was consistent across all sites and individuals, indicating that *A. palliata* relies on the combined use of these strategies for thermoregulation.

Across all study sites, howler monkeys varied their use of postures and tree strata in relation to ambient temperature. Notably, individuals adopting the extended posture in the lower stratum—typically under warmer conditions—rested for significantly longer periods (Table 2). These findings support the view that adjustments in resting time, combined with posture and stratum use, enable howler monkeys to maintain thermal balance, particularly under warm conditions. Given their limited capacity for physiological thermoregulation (Best and Kamilar 2018; Thompson and Hermann 2024), these behavioral strategies may necessitate extended resting periods to be effective. Such prolonged rest is likely an adaptive strategy to conserve both energy and water, resources essential for thermoregulation (Eppley et al. 2017; Fuller et al. 2021; Giroux et al. 2023).

The semi-fetal posture also showed a strong correlation with ambient temperature and rest duration. This posture—characterized by curled limbs close to the body—conserves heat and protects thermally vulnerable areas such as the ventral surface, which has less fur coverage (Paterson 1981; Bicca-Marques and Calegaro-Marques 1998; Palestino-Sánchez et al. 2020). In cold conditions, individuals frequently adopt this posture in the high strata, where exposure to solar radiation may aid in heat gain (Gestich et al. 2014; Brividoro et al. 2023; Thompson and Hermann 2024).

However, the semi-fetal posture in higher strata did not correspond to longer rest durations. Although this posture was more common during cold conditions and in the middle and upper strata, the mean time spent resting was not significantly greater than when using the extended posture in the lower stratum. In cold environments, mammals often expend more energy to maintain body temperature (Rhoades and Bell 2012; Tattersall et al. 2012; Mitchell et al. 2018). Similar patterns have been observed in other primates, such as baboons (Hill, 2006) and gorillas (Kosheleff and Anderson, 2009), which tend to be more active as ambient temperatures decrease throughout the day, which could be related to our observations, but further studies are required to verify this.

Various studies have reported that mantled howler monkeys exhibit a daily activity pattern where resting behavior dominates their activity budgets, accounting for between 60% and 80% of their time (Serio-Silva 1995; Milton 1998; Estrada et al. 1999; Asensio et al. 2007; Cristobal-Askarate and Arroyo-Rodríguez 2007; Schreier et al. 2021). This is because although they have a folivorous-frugivorous diet, they lack a specialized gastrointestinal tract for the digestion of the fibrous material present in the leaves they consume (Janiak et al. 2019), and this time is required for to ferment, digest, and extract energy from of fiber and the structural carbohydrates present in the leaves they consume (Milton 1981). However, our results demonstrate that ambient temperature has a significant influence on the thermal behavior and the resting duration of *A. palliata*. Although ecological factors such as food availability were not analyzed in this study, our findings support the hypothesis that thermal conditions play a more prominent role in shaping activity patterns than other ecological variables (Hilário et al. 2022). Although we only reported it in resting behavior.

Our findings indicate that mantled howler monkeys in the Los Tuxtlas region integrate body posture and tree stratum space use as part of a combined thermoregulatory strategy. As ambient temperatures rise, individuals increasingly adopt heat-dissipating behaviors—most notably the extended posture in the lower forest strata—while concurrently increasing rest duration, potentially as a means to reduce metabolic heat production and avoid hyperthermia. However, caution is warranted in interpreting these results. In this study, ambient temperature was recorded at a single vertical height, which may not accurately reflect the microclimatic conditions experienced by the monkeys across different canopy levels. Additionally, the study sites were relatively small (<20 ha), and habitat size can influence thermal dynamics; smaller forest fragments tend to exhibit reduced thermal buffering and more abrupt temperature fluctuations compared with continuous forest (Ewers and Banks-Leite 2013; Hofmeister et al. 2019). These factors may, in turn, influence the expression of thermoregulatory behaviors observed in this population.

Now, considering the continued increases in ambient temperature (Haustein et al. 2017; IPCC, 2023) may lead to a greater reliance on behavioral inactivity as a thermoregulatory strategy in *A. palliata*. While such responses may mitigate heat stress in the short term, prolonged increases in resting time during the active period may potentially reduce opportunities for essential behaviors such as foraging, feeding, and social interaction, potentially compromising individual fitness and long-term population viability. In addition, given that *A. palliata* primarily meets its hydration needs through food intake (Glander 1978; Nagy and Milton 1979), any disruption to feeding behavior caused by thermal stress may increase the risk of dehydration—a condition that has already been documented in this species, including mortality due to heat stroke (Pozo-Montuy et al. 2024). These findings underscore the importance of examining behavioral flexibility and thermal behavior in response to rising thermal stress, as understanding the limits and potential of such plasticity is critical for predicting the species’ capacity to cope with current thermal conditions.

## Supplementary Material

zoaf068_Supplementary_Data
