# Supplementary material for: Howler monkey (Alouatta palliata) thermal behavioral strategies and resting time are temperature-dependent
Source: Curr Zool. 2025 Oct 22;72(4):512–22. doi: 10.1093/cz/zoaf068 (PMC13500170; doi:10.1093/cz/zoaf068)
Supplement: Supplementary file 1 [file zoaf068_supplementary_data.zip › Appendix A.docx]

**Appendix A.** Matrices used in canonical correlation analyses

**Agaltepec (Aga)**

| Temperature | | | |
| --- | --- | --- | --- |
| Aga | 1. Low | 2. Middle | 3. High |
| 1. Semi-fetal | - | - | 20.79 |
| 2. Sitting | 33.25 | 27.03 | 25.19 |
| 3. Lying | 31.90 | 27.84 | 26.24 |
| 4. Spread | 32.28 | 31.79 | 28.61 |
| 5. Extended | 35.65 | 33.64 | - |
| Time | | | |
| Aga | 1. Low | 2. Middle | 3. High |
| 1. Semi-fetal | - | - | 37.13 |
| 2. Sitting | 12.06 | 18.61 | 14.39 |
| 3. Lying | 60.00 | 25.00 | 29.60 |
| 4. Spread | 30.06 | 31.12 | 28.93 |
| 5. Extended | 54.78 | 49.17 | - |

**Mirador Pilapa (MP)**

| Temperature | | | |
| --- | --- | --- | --- |
| MP | 1. Low | 2. Middle | 3. High |
| 1. Semi-fetal | - | 27.65 | 27.12 |
| 2. Sitting | 30.06 | 29.21 | 28.11 |
| 3. Lying | 27.73 | 28.41 | 28.63 |
| 4. Spread | 29.11 | 29.52 | 29.65 |
| 5. Extended | 30.88 | 30.40 | - |
| Time | | | |
| MP | 1. Low | 2. Middle | 3. High |
| 1. Semi-fetal | - | 8.08 | 8.22 |
| 2. Sitting | 4.27 | 4.25 | 4.38 |
| 3. Lying | 7.56 | 8.77 | 8.23 |
| 4. Spread | 8.50 | 8.57 | 8.27 |
| 5. Extended | 9.18 | 8.92 | - |

**Zapoapan (Zap)**

| Temperature | | | |
| --- | --- | --- | --- |
| Zap | 1. Low | 2. Middle | 3. High |
| 1. Semi-fetal | - | 24.90 | 24.83 |
| 2. Sitting | 27.98 | 28.18 | 28.81 |
| 3. Lying | 28.60 | 27.85 | 27.96 |
| 4. Spread | 29.84 | 29.90 | 29.78 |
| 5. Extended | 30.98 | 31.18 | - |
| Time | | | |
| Zap | 1. Low | 2. Middle | 3. High |
| 1. Semi-fetal | - | 7.33 | 7.92 |
| 2. Sitting | 3.06 | 3.85 | 3.34 |
| 3. Lying | 8.83 | 8.66 | 8.76 |
| 4. Spread | 8.25 | 8.69 | 7.91 |
| 5. Extended | 9.31 | 9.08 | - |
