# Supplementary material for: Howler monkey (Alouatta palliata) thermal behavioral strategies and resting time are temperature-dependent
Source: Curr Zool. 2025 Oct 22;72(4):512–22. doi: 10.1093/cz/zoaf068 (PMC13500170; doi:10.1093/cz/zoaf068)
Supplement: Supplementary file 1 [file zoaf068_supplementary_data.zip › Appendix B.docx]

**Appendix B.** Multiple comparisons of the temperature and time recorded in each posture during the rest of *A. palliata*

**Agaltepec (Aga)**

Temperature vs Posture

Kruskal-Wallis chi-squared = 120.1, df = 4, p-value < 2.2e-16

Pairwise comparisons using Wilcoxon rank sum test with continuity correction

|  | Semi-fetal | Sitting | Lying | Spread |
| --- | --- | --- | --- | --- |
| Sitting | 1.8e-07 | - | - | - |
| Lying | 4.9e-08 | 0.47 | - | - |
| Spread | 8.9e-12 | 0.00089 | 1.2e-05 | - |
| Extended | 4.6e-12 | 5.7e-14 | 7.7e-16 | 5.8e-10 |

Time vs Posture

Kruskal-Wallis chi-squared = 153.72, df = 4, p-value < 2.2e-16

Pairwise comparisons using Wilcoxon rank sum test with continuity correction

|  | Semi-fetal | Sitting | Lying | Spread |
| --- | --- | --- | --- | --- |
| Sitting | 1.4e-12 | - | - | - |
| Lying | 0.0015 | 6.1e-14 | - | - |
| Spread | 0.0153 | <2e-12 | 0.33438 | - |
| Extended | 0.0094 | <2e-16 | 9.1e-10 | 9.1e-10 |

**Mirador Pilapa (MP)**

Temperature vs Posture

Kruskal-Wallis chi-squared = 460.34, df = 4, p-value < 2.2e-16

Pairwise comparisons using Wilcoxon rank sum test with continuity correction

|  | Semi-fetal | Sitting | Lying | Spread |
| --- | --- | --- | --- | --- |
| Sitting | <2e-16 | - | - | - |
| Lying | <2e-16 | 0.11 | - | - |
| Spread | <2e-16 | 6.6e-09 | <2e-16 | - |
| Extended | <2e-16 | <2e-16 | <2e-16 | <2e-16 |

Time vs Posture

Kruskal-Wallis chi-squared = 481.55, df = 4, p-value < 2.2e-16

Pairwise comparisons using Wilcoxon rank sum test with continuity correction

|  | Semi-fetal | Sitting | Lying | Spread |
| --- | --- | --- | --- | --- |
| Sitting | <2e-16 | - | - | - |
| Lying | 0.10445 | <2e-16 | - | - |
| Spread | 0.20036 | <2e-16 | 0.69592 | - |
| Extended | 7.1e-06 | <2e-16 | 0.00119 | 0.00028 |

**Zapoapan (Zap)**

Temperature vs Posture

Kruskal-Wallis chi-squared = 899.08, df = 4, p-value < 2.2e-16

Pairwise comparisons using Wilcoxon rank sum test with continuity correction

|  | Semi-fetal | Sitting | Lying | Spread |
| --- | --- | --- | --- | --- |
| Sitting | <2e-16 | - | - | - |
| Lying | <2e-16 | 2.9e-08 | - | - |
| Spread | <2e-16 | <2e-16 | <2e-16 | - |
| Extended | <2e-16 | <2e-16 | <2e-16 | <2e-16 |

Time vs Posture

Kruskal-Wallis chi-squared = 676.11, df = 4, p-value < 2.2e-16

Pairwise comparisons using Wilcoxon rank sum test with continuity correction

|  | Semi-fetal | Sitting | Lying | Spread |
| --- | --- | --- | --- | --- |
| Sitting | <2e-16 | - | - | - |
| Lying | 0.00053 | <2e-16 | - | - |
| Spread | 0.02050 | <2e-16 | 0.10493 | - |
| Extended | 8.9e-08 | <2e-16 | 0.04564 | 2.8e-05 |
