# Supplementary material for: Howler monkey (Alouatta palliata) thermal behavioral strategies and resting time are temperature-dependent
Source: Curr Zool. 2025 Oct 22;72(4):512–22. doi: 10.1093/cz/zoaf068 (PMC13500170; doi:10.1093/cz/zoaf068)
Supplement: Supplementary file 1 [file zoaf068_supplementary_data.zip › Appendix C.docx]

**Appendix C.** Multiple comparisons of the temperature and time recorded in each tree strata during the rest of *A. palliata*

**Agaltepec (Aga)**

Temperature vs Posture

Kruskal-Wallis chi-squared = 120.02, df = 2, p-value < 2.2e-16

Pairwise comparisons using Wilcoxon rank sum test with continuity correction

|  | Low | Middle |
| --- | --- | --- |
| Middle | 6.3e-10 | - |
| High | <2e-16 | 7.3e-16 |

Time vs Posture

Kruskal-Wallis chi-squared = 12.49, df = 2, p-value = 0.00194

Pairwise comparisons using Wilcoxon rank sum test with continuity correction

|  | Low | Middle |
| --- | --- | --- |
| Middle | 0.00429 | - |
| High | 0.00056 | 0.26477 |

**Mirador Pilapa (MP)**

Temperature vs Posture

Kruskal-Wallis chi-squared = 291.86, df = 2, p-value < 2.2e-16

Pairwise comparisons using Wilcoxon rank sum test with continuity correction

|  | Low | Middle |
| --- | --- | --- |
| Middle | <2e-16 | - |
| High | <2e-16 | <2e-16 |

Time vs Posture

Kruskal-Wallis chi-squared = 41.699, df = 2, p-value = 8.813e-10

Pairwise comparisons using Wilcoxon rank sum test with continuity correction

|  | Low | Middle |
| --- | --- | --- |
| Middle | 1.4e-05 | - |
| High | 2.6e-10 | 0.0022 |

**Zapoapan (Zap)**

Temperature vs Posture

Kruskal-Wallis chi-squared = 364.41, df = 2, p-value < 2.2e-16

Pairwise comparisons using Wilcoxon rank sum test with continuity correction

|  | Low | Middle |
| --- | --- | --- |
| Middle | 9.4e-16 | - |
| High | <2e-16 | <2e-16 |

Time vs Posture

Kruskal-Wallis chi-squared = 48.126, df = 2, p-value = 3.544e-11

Pairwise comparisons using Wilcoxon rank sum test with continuity correction

|  | Low | Middle |
| --- | --- | --- |
| Middle | 5.0e-05 | - |
| High | 1.3e-11 | 0.00014 |
