# Supplementary material for: Howler monkey (Alouatta palliata) thermal behavioral strategies and resting time are temperature-dependent
Source: Curr Zool. 2025 Oct 22;72(4):512–22. doi: 10.1093/cz/zoaf068 (PMC13500170; doi:10.1093/cz/zoaf068)
Supplement: Supplementary file 1 [file zoaf068_supplementary_data.zip › Appendix-D.docx]

**Appendix D.** Maximize correlation between transformed variables time and environmental temperature recorded in each posture in tree strata. The purple color corresponds to the temperature data recorded in each thermal strategy, and the blue color corresponds to the time data recorded in each thermal strategy. The ellipse indicates the relationship between posture and arboreal strata.
